# Spontaneous Disappearance of Arachnoid Cyst after Head Trauma

**DOI:** 10.5334/jbr-btr.847

**Published:** 2015-09-15

**Authors:** L. Morbée, Ph. Lagae, P. Jeannin, N. Baelde, J. De Mey

**Affiliations:** 1 Department of Radiology, University Hospital, Brussels, Belgium; 2 Department of Radiology, AZ Jan Palfijn, Ghent, Belgium

A 4-year-old boy was referred to the pediatrician for an increased head circumference, noted to be in the 95th percentile. His neurological examination showed no focal neurological abnormality, cranial nerve deficit or papilledema. MRI of the brain (Fig. A) revealed a well-defined, nonenhancing, extra-axial mass in the right frontotemporal region, between the tabula interna and the insula. The mass is isointense to CSF on T1 (Figs. A, B) and T2 weighted images (Fig. C). This fluid collection suppresses completely with FLAIR and shows no restriction on DWI. There is scalloping of bone margins and medial displacement of cortical veins. Based on these imaging findings the diagnosis of a right frontotemporal arachnoid cyst was made.

Five years later the patient experienced a painful, hard blow against a lamppost on the right side of the head. Five weeks after this head trauma a follow-up MRI of the brain was performed (Figs. D–F). Unexpectedly, the arachnoid cyst has regressed for the most part. Most likely, resorption of the cyst has occurred after the head trauma. There is a little linear collection of fluid in the subdural region, which is isointense on T1 weighted images (Fig. D), hyperintense on T2 weighted images (Fig. E) without suppression on FLAIR (Fig. F). T2* weighted images show no evidence of late subacute hemorrhage. Probably, there is little granulation tissue formation in reaction after trauma which causes relatively high protein content in this subdural fluid collection.

**Figures A–F:**
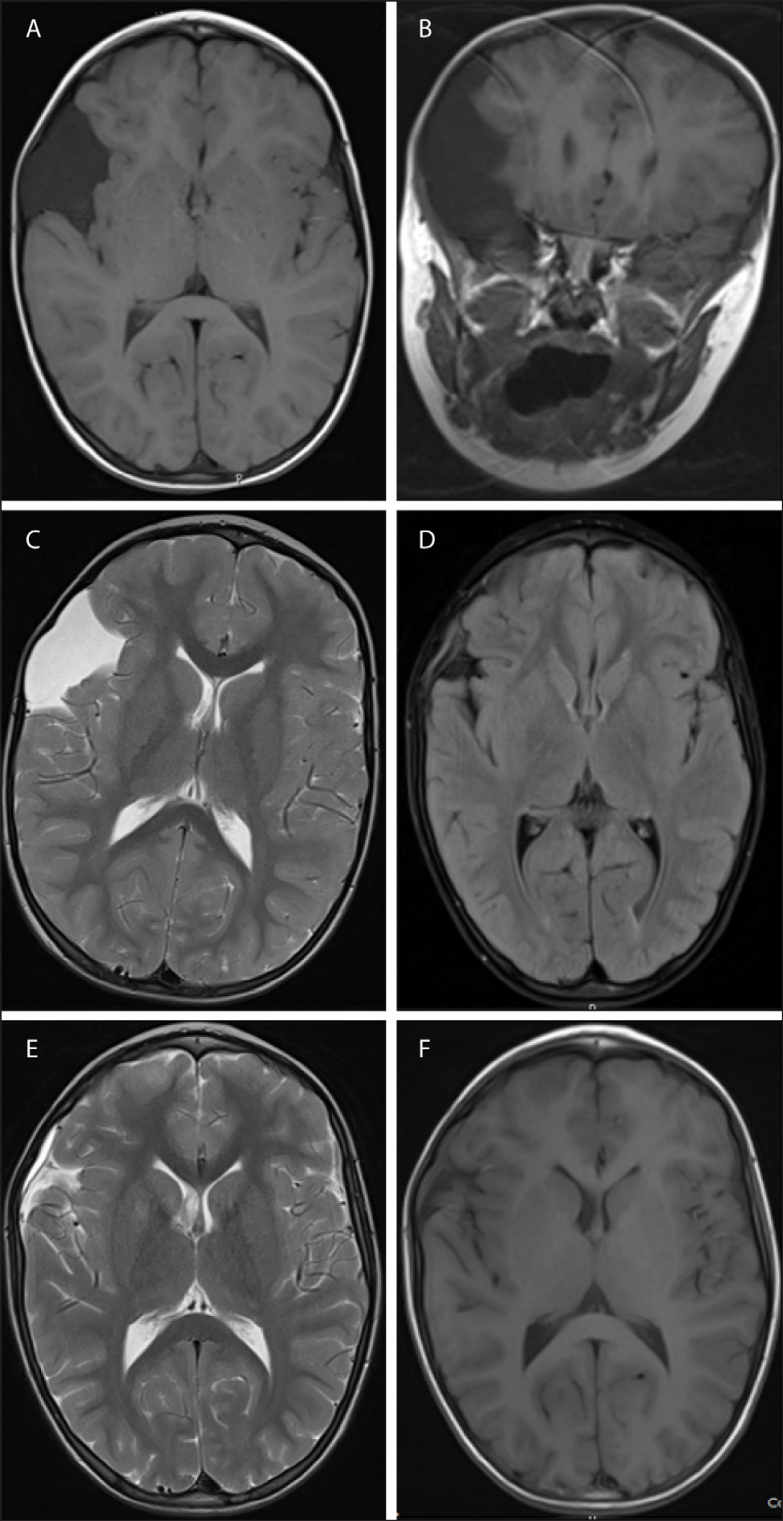


## Comment

Arachnoid cysts are benign lesions that occur in association with the central nervous system, both within the intracranial compartment (most common) as well as within the spinal canal. They are usually located in the subarachnoid space and account for 1% of all intracranial masses. The frontotemporal region is the most commonly affected.

The majority of arachnoid cysts are asymptomatic, even when the cysts become quite large. When symptoms do occur, they are usually the result of gradual enlargement resulting in mass effect: headache, calvarial bulging and seizures. Controversy surrounds the treatment of these cysts. Some clinicians advocate treating only patients with symptomatic cysts, whereas others believe that even asymptomatic cysts should be decompressed to avoid future complications.

In this case the most recent MRI reveals spontaneous regression of the cyst after head trauma. This suggests a possible mechanism by which this type of cyst (associated with subdural effusion) might disappear. The rupture of the cyst wall appears to be essential for the arachnoid cyst to disappear. After rupture, subdural effusion must develop around the cyst. As this effusion is absorbed, the fluid in the cyst drains away, after which the cyst becomes smaller and gradually disappears. This supports the possibility of a “natural cure” for arachnoid cysts without surgical intervention.

MRI is the imaging study of choice in the detection of arachnoid cysts because of its ability to demonstrate the exact location, extent and relationship of the arachnoid cyst to adjacent brain or spinal cord.

An important mimic of an arachnoid cyst is an epidermoid cyst. However, epidermoid cysts show restricted diffusion on DWI and give a more heterogenous and higher signal on FLAIR imaging. The differential diagnosis also includes chronic subdural hematoma, which is often bilateral and may show an enhancing membrane and a signal that is not identical to CSF. A subdural hygroma is also often bilateral and has a crescenteric or flat configuration. Other nonneoplastic cysts include porencephalic cysts, neurenteric cysts and neuroglial cysts. The latter two are rare. Porencephalic cysts are surrounded by gliotic brain and not compressed cortex. Cystic tumours, such as pilocytic astrocytoma and haemangioblastoma, often will have a solid, enhancing component and be intra-axial.

## Competing Interests

The authors declare that they have no competing interests.
